# Reduced plasma nicotinamide level and altered NAD^+^ metabolism in glial cells surrounding Aβ plaques in the mouse model of Alzheimer’s disease

**DOI:** 10.1002/alz.088847

**Published:** 2025-01-03

**Authors:** Michiko Sekiya, Yasufumi Sakakibara, Yu Hirota, Naoki Ito, Kimi Takei, Sachie Chikamatsu, Risa Nishijima, Koichi M. Iijima

**Affiliations:** ^1^ Nagoya City University, Nagoya Japan; ^2^ National Center for Geriatrics and Gerontology, Obu Japan; ^3^ Japan Society for the Promotion of Science, Chiyoda‐ku Japan; ^4^ National Center for Geriatrics and Gerontology, Obu, Aichi Japan

## Abstract

**Background:**

Alzheimer’s disease (AD) is a progressive neurodegenerative disease and a leading cause of senile dementia. Accumulation of amyloid‐β (Aβ) in the brains causes chronic neuroinflammation, synaptic loss, and neurovascular damage, which is thought to initiate decades‐long AD pathogenesis. Recent clinical trials for anti‐Aβ immunotherapy highlights the utility of biomarkers that faithfully reflect Aβ‐related brain pathology to diagnose AD at the preclinical stage, to predict the onset and progression of the disease, and to assess the therapeutic efficacy of drugs.

**Method:**

To search for plasma metabolites reflecting neuroinflammation due to Aβ accumulation, we conducted untargeted metabolomics in plasma and analyzed potential roles of metabolic changes in neuroinflammatory response in brains using *App*knock‐in (*App^NLGF^*) mouse model of brain parenchymal Aβ amyloidosis.

**Result:**

The capillary electrophoresis‐time‐of‐flight mass spectrometry analysis on plasma samples from wild‐type and *App^NLGF^* mice revealed that plasma nicotinamide levels were significantly reduced in *App^NLGF^* mice, and “nicotinate and nicotinamide metabolism” was the most enriched pathway of altered metabolite profiles. Nicotinamide is a precursor of nicotinamide adenine dinucleotide (NAD^+^). In *App^NLGF^* mouse brains, while NAD^+^ levels were unaltered, mRNA levels of NAD^+^‐synthesizing and NAD^+^‐degrading genes were increased, and these enzymes were expressed in reactive astrocytes and microglia surrounding Aβ plaques.

**Conclusion:**

This study suggests that alterations in plasma nicotinamide levels may be a potential biomarker to assess neuroinflammatory response against Aβ pathology and “nicotinate and nicotinamide metabolism” may be a potential therapeutic target to prevent the onset of AD by targeting both neuroinflammation and neuroprotection.

